# Impact of Advance Care Planning on the Hospitalization-Associated Utilization and Cost of Patients with Alzheimer’s Disease-Related Disorders Receiving Primary Care via Telehealth in a Provider Shortage Area: A Quantitative Pre-Study

**DOI:** 10.3390/ijerph20126157

**Published:** 2023-06-16

**Authors:** Ji Won Yoo, Peter S. Reed, Jay J. Shen, Jennifer Carson, Mingon Kang, Jerry Reeves, Yonsu Kim, Ian Choe, Pearl Kim, Laurie Kim, Hee-Taik Kang, Maryam Tabrizi

**Affiliations:** 1 Department of Internal Medicine, Kirk Kerkorian School of Medicine at UNLV, Las Vegas, NV 89154, USA; 2 Sanford Center for Aging, Reno School of Medicine, University of Nevada, Reno, NV 89557, USA; 3 School of Public Health, University of Nevada, Reno, NV 89557, USA; 4 School of Public Health, University of Nevada, Las Vegas, NV 89119, USAyonsu.kim@unlv.edu (Y.K.);; 5 Department of Computer Science, Howard Hughes College of Engineering, University of Nevada, Las Vegas, NV 89154, USA; 6 Comagine Health, Las Vegas, NV 89118, USA; 7 Telehealth Divison, Optum Care Network of Nevada, Las Vegas, NV 89128, USA; ian.choe@optum.com; 8 Department of Family Medicine, Yonsei University College of Medicine, Seoul 03722, Republic of Korea; 9 Department of Clinical Sciences, School of Dental Medicine, University of Nevada, Las Vegas, NV 89154, USA

**Keywords:** advance care plan, age-friendly health system, dementia, finances, primary care, telehealth

## Abstract

Telehealth has been adopted as an alternative to in-person primary care visits. With multiple participants able to join remotely, telehealth can facilitate the discussion and documentation of advance care planning (ACP) for those with Alzheimer’s disease-related disorders (ADRDs). We measured hospitalization-associated utilization outcomes, instances of hospitalization and 90-day re-hospitalizations from payors’ administrative databases and verified the data via electronic health records. We estimated the hospitalization-associated costs using the Nevada State Inpatient Dataset and compared the estimated costs between ADRD patients with and without ACP documentation in the year 2021. Compared to the ADRD patients without ACP documentation, those with ACP documentation were less likely to be hospitalized (mean: 0.74; standard deviation: 0.31; *p* < 0.01) and were less likely to be readmitted within 90 days of discharge (mean: 0.16; standard deviation: 0.06; *p* < 0.01). The hospitalization-associated cost estimate for ADRD patients with ACP documentation (mean: USD 149,722; standard deviation: USD 80,850) was less than that of the patients without ACP documentation (mean: USD 200,148; standard deviation: USD 82,061; *p* < 0.01). Further geriatrics workforce training is called for to enhance ACP competencies for ADRD patients, especially in areas with provider shortages where telehealth plays a comparatively more important role.

## 1. Introduction

Telehealth services enhance access to primary care for older and vulnerable adults, especially for those needing transportation support [1,2,3]. Studies on telehealth’s impact on health outcomes have shown that telehealth utilization is associated with better chronic disease management and reduces healthcare disparities [4,5,6]. The emergence of the Coronavirus disease 19 (COVID-19) pandemic highlighted the need for telehealth for these individuals [7,8]. The CARES Act led to the rapid implementation of telehealth across the United States (USA).

During the COVID-19 pandemic, many primary care and public transportation services were severely restricted due to public health concerns and lack of staff. Such restricted access posed special challenges for people living with Alzheimer’s disease-related disorders (ADRD), who often require increased support through care coordination and partnership with healthcare providers, caregivers and community stakeholders. Hence, telehealth provided opportune leverage in the care of the people living with ADRD. In response to the pandemic, the State of Nevada established a statewide platform for integrating health system and community services through the Pathways Community HUB: a model for the coordination of community health care [9]. This effort was particularly germane in the State of Nevada, where the number of primary care providers per 100,000 populations is the lowest among the 50 states (205.1 vs. a US average of 265.8 in 2022) [10]. In early 2020, the Nevada Aging and Disability Services Division, Nevada’s state unit for aging, launched the Nevada COVID-19 Aging Network Rapid Response (Nevada CAN) to mobilize a statewide response to ensure homebound elders retained access to food and medications, social support and telehealth services. Nevada CAN included the state’s two Geriatrics Workforce Enhancement Programs (GWEPs) at the University of Nevada, Reno, and the University of Nevada, Las Vegas (UNLV), which engaged primary care provider organizations alongside community-based aging service organizations to establish statewide telehealth network, the Nevada Geriatrics Telehealth Collaborative (NGTC), with the ultimate goal of ensuring access to healthcare for community-dwelling older adults during the unprecedented COVID-19 pandemic [11]. 

More than 6 million Americans live with ADRD [12]. Systems of care not well aligned to meet the needs of those who are affected by ADRD. Half of the individuals with ADRD do not receive a diagnosis, and their caregiving needs heavily rely on informal caregivers and care partners [12]. In the recent Agency for Healthcare Research and Quality systematic review, the overwhelming majority were disconnected from the delivery of system care for ADRD, which then led to low-value care with high burdens of healthcare and societal costs [13]. Due at least in part to this widespread disconnect, the estimated annual cost of Medicare beneficiaries with ADRD (USD 43,444) was approximately three times the cost of those without ADRD (USD 14,593) in 2022 [12]. To leverage a reconnection between the health system and informal caregiving supports for people living with ADRD, advance care planning (ACP) documentation, with discussions occurring within at least 2 years of ADRD diagnosis, has been recommended as a dementia management quality measure [12,13,14]. ACP documentation plays the critical role of integrating health information across care settings and partners to better align initiatives, striving to transform clinical care in partnership with community stakeholders [15]. The Center for Medicare and Medicaid Services (CMS) designated the ACP documentation for patients aged 65 and older as a Merit-Based Incentive Payment System (MIPS) quality measure in ambulatory care [13]. Although the CMS reimburses healthcare providers at a rate of USD 80–86 for the first 30 min and USD 75 for each 30 min thereafter, ACP discussion has been underutilized and even under-claimed for Medicare beneficiaries. For example, data show that 1.5% of eligible Medicare beneficiaries were billed for ACP discussion in 2019 [16]. Such low ACP claim rates notwithstanding, considerable amounts of revenue can be generated by providers who do proactively provide and document ACP appropriately in practice. 

Nevada is the state with the third fastest growing number of people living with ADRD per capita among states in the USA [12]. Further, Nevada ranks highest in terms of 30-day hospital readmission rates (25.8%), and in 2018, it was the fifth highest state in terms of ADRD care expenditures due to ED visits and hospital readmissions per Medicare beneficiaries with ADRD [12]. Potential causes of these high-cost burdens of ADRD care in Nevada have not been explored. While one cause would likely be the widespread lack of ACP and the documentation thereof, its impact on hospitalization-associated utilization and concomitant healthcare costs is largely unknown. The impact of ACP documentation on hospitalization-associated utilization and healthcare costs is largely unknown. In a recent study at a single tertiary academic medical center, ACP completion was associated with higher rates of care de-escalation and conversion from life-sustaining treatment to comfort care, for example, withholding futile feeding tubes for caring for people living with advanced ADRD [17]. ACP documentation among people living with ADRD may facilitate not only high-value care but also efficient care delivery to avoid unnecessary hospitalizations and to expedite hospital discharge planning [17,18]. The primary purpose of the study was to evaluate the impact of ACP documentation on hospitalization-associated utilization and the concomitant costs among patients diagnosed with ADRD. Another purpose of the study was to measure trends of ACP billing for patients diagnosed with ADRD before and after primary care telehealth visits.

## 2. Materials and Methods

### 2.1. Design Overview and Study Sample

**Figure 1:**
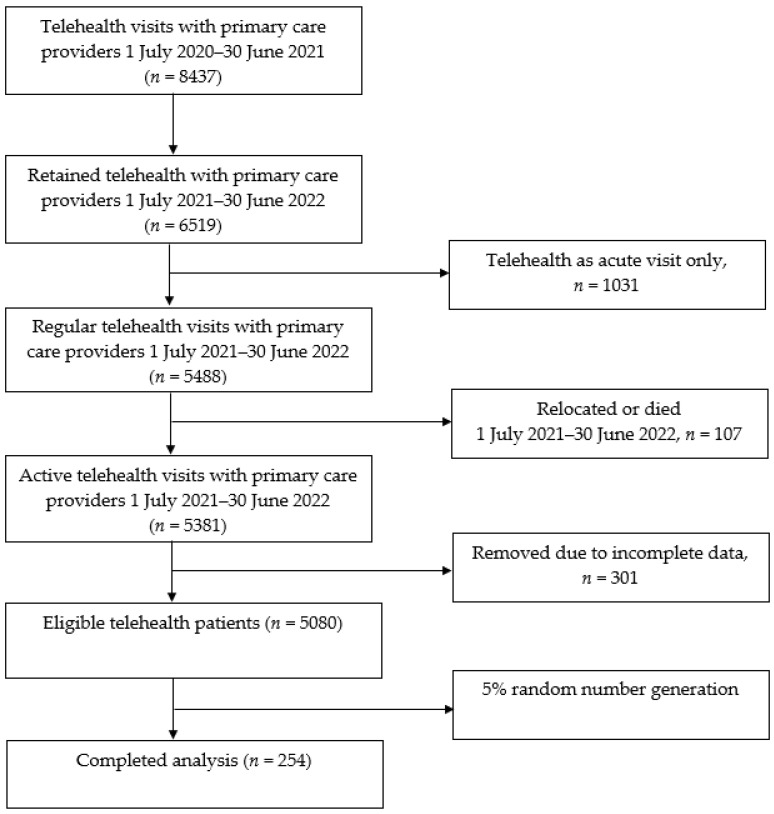
Recruitment flow diagram.

This was a retrospective, cross-sectional study of Medicare Advantage enrollees seen by primary care providers in an urban non-for-profit organization, seven primary care sites and over 100 providers in the Southwest. While available prior to the COVID-19 pandemic, the frequency of telehealth service delivery increased significantly, with widespread adoption rolling out in March 2020 as a response to the public health emergency declaration. Figure 1 depicts the process of participant inclusion [19]. The electronic health records (EHRs) of 8960 patients with video telehealth visits between 1 January 2020 and 31 December 2020 were collected, including patient demographics and the dates of services. Seven thousand one hundred twenty-one patients were seen in telehealth primary care between 1 January 2021 and 31 December 2021. Nine hundred twelve patients were excluded because their visits were acute in nature only, precluding ACP discussion and the documentation thereof. Three hundred and sixty-seven patients were removed due to either death or departure from the aforementioned health system. Among the 5505 patients with active telehealth primary care visits, 367 patients were removed due to incomplete data, and 5032 patients were excluded due to the absence of dementia according to the International Classification of Diseases, Tenth Revision, Clinical Modification (ICD-10-CM codes: either F01, F02, or F03). Among the 473 patients with dementia per the ICD-10-CM codes, 58 patients were collected when either ACP Current Procedural Terminology (CPT) codes 99497 or 99498 were documented. Among the remaining 415 patients without either of the codes, 58 patients were selected using the multivariate distant matching (MDM) process [20]. Age, gender, race and Charlson Comorbidity Index (CCI) variables were used to apply the MDM process [20]. We estimated that a sample size of 57 was required from the preliminary analysis comparing hospitalization-associated cost estimates between patients with (mean: USD 143,865) and without ACP (mean: USD 197,384; standard deviation: USD 101,914), with a power of 80% and an alpha of 5% [21].

### 2.2. Outcomes and Measurable Variables

The main outcome of interest had three aspects: (1) hospitalization-associated utilization, (2) hospitalization-associated cost and (3) trends in ACP billing rates before and after the implementation of telehealth primary care. Hospitalization-associated utilization information was collected from the Medicare Advantage payor programs. We also verified this information by reviewing the hospital discharge summaries, including the principal diagnoses and hospital length of stay information from the EHR. The hospitalization-associated cost was estimated from the Nevada State Inpatient Database (SID) between 1 January and 31 December 2021, which is a publicly available dataset [22]. The Nevada SID contains hospital discharge records of all community hospitals in the state of Nevada, and it was originally developed for the Healthcare Cost and Utilization Project (HCUP) by AHRQ. The Nevada SID covers more than 95% of all Nevada hospital discharges [22]. The Nevada SID files were constructed from hospital discharge files received from the UNLV Center for Health Information Analysis (CHIA) under the authority of the Nevada Division of Healthcare Financing and Policy (DHCFP) [21]. The Nevada SID includes anonymous patient-level information including demographics, diagnostic/procedure codes and hospital utilizations. We collected hospitalization lengths of stay (days) and hospital charges per principal diagnoses from the Nevada SID. Then, the hospitalization-associated cost was estimated by combining the hospital lengths of stay (days) and the daily average hospital charges per principal diagnoses. Hospitalization-associated cost estimates were weighted by age and gender. ACP billing was defined as either ACP 99497 or 99498 among ADRD patients (*n* = 473), who were identified via ICD-10-CM codes (either F01, F02, or F03) in EHR. The rate of ACP billing was defined as each ACP 99497 and 99498 per ADRD patient (*n* = 473). As telehealth primary care was adopted in 2020, the trends in the ACP billing rates of ADRD patients were the difference of the rates before (1 January–31 December 2019) and after (1 January–31 December 2021) the implementation of primary telehealth healthcare. The measurable variables were age, race, gender and weighted CCI [23]. Age was automatically generated from the participant’s date of birth to the data access date, 1 January 2021. Age, race and gender information were extracted from the EHR administrative dataset. Race data were self-reported. Race was divided into non-Hispanic White, non-Hispanic Black, Hispanic, non-Hispanic Asian and other or mixed races. The CCI was calculated as a measure of comorbidity [23].

### 2.3. Statistical Analyses

We examined the descriptive statistics of a patient’s characteristics by calculating means, standard deviations and frequencies for each variable. 

To test the (1) hospitalization-associated utilization outcomes (number of hospitalization and 90-days admission) between ADRD patients with and without ACP documentation and (2) the hospitalization-associated cost estimate between ADRD patients with and without ACP documentation, paired *t*-tests were used. To test the trends in the ACP billing rates of ADRD patients before and after the implementation of telehealth primary care, the χ^2^ test was used. All statistical analyses were two-tailed, and a *p*-value less than 0.05 was statistically significant. STATA, version 17 (Stata Corp, College Station, TX, USA), was used for statistical analysis. This work was determined to be quality improvement/evaluation by the UNLV Institutional Review Board (IRB), and therefore not subject to IRB approval and oversight as human subject research.

## 3. Results

Of the 58 patients diagnosed with ADRD, described in Table 1, the mean age was 81.3 years, with a standard deviation if 9.7 years and a range of 61–98 years. Slightly more than one-third (37.9%) were younger than 79 years. In terms of participant race/ethnicity, 51.7% were non-Hispanic White, 15.5% were non-Hispanic Black, 13.8% were Hispanic, 12.1% were non-Hispanic Asian and 5.9% were of other or mixed races; 41.4% were male, 55.3% were female, and 3.6% were other; the mean CCI was 3.9, with a standard deviation of 2.7 and a range of 2–11.

### Tables

As shown in Table 2, the hospitalization-associated utilization outcomes were compared between ADRD patients with or without ACP documentation. Compared to the patients without ACP documentation, those with ACP documentation were less likely to hospitalized (mean: 0.74; standard deviation: 0.31; *p* < 0.01) and were less likely to be readmitted within 90 days of discharge from the hospital (mean: 0.16; standard deviation: 0.06; *p* < 0.01).

Table 3 demonstrates the estimated hospitalization-associated cost between patients with and without ACP documentation. The cost estimate of patients with ACP documentation (mean: USD 149,722; standard deviation: USD 80,850) was less than that of patients without ACP documentation (mean: USD 200,148; standard deviation: USD 82,061: *p* < 0.01).

Table 4 demonstrates the trends in the ACP billing rates of patients diagnosed with ADRD before and after the implementation of telehealth primary care. Documentation of ACP billing code 99,498 was not identified either before or after the implementation of telehealth primary care. The ACP billing rate after the implementation of telehealth primary care (12.3%; 58/473) was higher than the rate before implementation (0.8%, 4/473; χ^2^ = 50.3; *p* < 0.001).

## 4. Discussion

The purpose of the study is to better understand the impact of ACP discussion and documentation thereof on healthcare-associated utilization and its concomitant cost among patients with ADRD receiving telehealth primary care. To the best of our knowledge, this is the first report affirming the role of telehealth primary care for ADRD patients in enhancing ACP discussion and the documentation thereof. The results revealed that ACP documentation was associated with fewer hospitalizations and 90-day readmissions. Unsurprisingly, higher ACP billing rates were also associated with lower hospitalization-associated costs. These findings corroborate the hypothesis that ACP documentation facilitated care coordination and efficient care delivery as well as saved healthcare costs [12,13,17,18]. In the midst of the payment system transition of Medicare beneficiaries from fee-for-service payments to alternative payment systems (for example, bundle payments), these findings highlight opportunities for reforming provider payments to achieve greater equity and value in providing needed care to people living with ADRD [24]. These findings might go so far as to provide evidence that savings in Medicare Part A can be achieved via the providers’ endeavors in Medicare Part B [24]. Notwithstanding such benefits, less than 13% of the opportunities were utilized for ACP practice and the documentation thereof, as revealed by our analysis. While the overall trends have an upward momentum when compared with the year prior, the low rates of ACP claims are also seen in a nationwide analysis of claims data [16]. 

Several qualitative studies exploring the low rate of ACP billing suggested insights. Numerous training and awareness programs have not successfully led to change among healthcare providers, especially in terms of primary care providers’ behaviors to enhance ACP documentation and billing [25,26,27,28]. As the burden of EHR inbox messages and alerts has emerged as a noteworthy source of physician burnout in recent years, ACP documentation alerts in EHR may likely contribute as an additional source [28,29]. Consequently, providers likely dismiss ACP alerts in much the same way that they dismiss others when awash with alerts at all times [28,29]. Indeed, alert fatigue is a typical form of dismissal bias [29]. Dismissal bias arises from a gradual desensitization to alerts, especially when alerts are not correctly updated in the health system [29]. 

Furthermore, there are other factors in the aversion of primary care providers to ACP documentation. Among the most poignant reasons, though not often discussed, is that the very effort to have ACP discussion with patients and their caregivers is often misconstrued as an alarm or even a warning. To that end, a validated predictive tool for a given ADRD patient’s transition from independence to nursing home dependence may greatly help in compelling the ACP discussion; however, such a tool is currently unavailable. For those patients with ADRD who are already nursing home residents, however, the Advanced Dementia Prognostic Tool (ADPT) can be used to prognosticate an estimated life expectancy of less than 6 months [30]. A nationwide effort is underway to provide helpful tools for healthcare providers; the Learning Health System (LHS) in ADRD care has been introduced with aim of harnessing data and analytics to drive objective clinical data transformation in cycles of continuous improvement for patients, caregivers, healthcare professionals and community stakeholders [13]. The LHS aligns the power of digital health to improve the outcomes of ADRD care in a harmony between the health system and the community [13]. Under the LHS paradigm, data collection, analysis using artificial intelligence, applications to the real world and real-time practice may enhance confidence in ADRD care planning [13]. Although ePrognosis has been widely used to predict mortality and disability for the past two decades from the self-report dataset, the Health and Retirement Study (HRS), the data were collected from a sample with an over-representation of non-Hispanic white subjects (85.7%) [31]. The paucity of racial and ethnic minorities in the data set encourages further efforts to both identify and eliminate bias within healthcare systems, which will help advance healthcare equity [32]. 

It is worth noting that with the widespread adaptation of machine learning and artificial intelligence, the adoption of algorithmic fairness principles has emerged as an important requirement for mitigating racial and ethnic bias [29]. This fairness algorithm enhances the integrity of fairness in clinical applications of machine learning by reducing the gap of predictive performance between racial and ethnic majority and minority groups [29].

Lastly, a call to action is needed to develop and sustain an infrastructure grounded in collaborative research and community engagement to respond to low rates of ACP documentation for the people living with ADRD [13]. Greater awareness of the potential and likely harm from the lack of ACP documentation will likely play a pivotal role in training a future geriatrics workforce to provide both telehealth and in-person primary care services [33]. 

In a provider shortage area, workforce training is a realistic priority strategy to enhance quality of care outcomes [34]. The Age-Friendly Health System 4M framework (What Matters, Mobility, Medication, Mentation) is an easily adoptable toolkit for evaluating the quality of care and a workforce education competency tool for use across diverse local healthcare systems and care settings [33,34,35]. In the recent quantitative analysis of reviewing EHRs to measure the rate of documenting 4M elements in one Nevada health system adopting the 4M framework in its primary telehealth services, What Matters (62.2%) was the most common 4M element to be documented, and within the components of What Matters, ACP discussion was the most commonly (29.0%) documented [36]. Although the cost-effectiveness for becoming an age-friendly health system via training an ADRD-aware workforce remains to be determined, adopting the 4M framework for people living with ADRD will not likely add significant cost burdens to a health system [33,35]. As such, the 4M framework can be a promising innovative strategy to enhance the outcomes of ADRD care in Nevada [37]. 

Finally, as telehealth has been adopted as a modality of care for people living with ADRD since the COVID-19 pandemic, it is essential to develop and support user-friendly health information technology for care partners [13]. This is especially important for timely communication with healthcare providers in transitional care coordination, for example, within two weeks of hospital discharge. In alignment with the current NGTC platform [11], which promotes the utilization of telehealth services for Nevada’s older adults, the Nevada Memory Network (NMN) is under review by the Nevada State Legislature [38]. If approved by the State Legislature in early 2023, the NMN can play a facilitative role in the early detection of ADRD in primary care, expedited referrals to specialists and community resources as well as ACP discussions with primary care providers and care partners [38]. The NMN will utilize telehealth as a core care service delivery tool by the NMN [38].

ADRD is a leading cause of death and disability globally. The estimated global economic costs of ADRD doubled in the recent decade, from USD 604 billion in 2010 to USD 1313 billion in 2019, corresponding to USD 23,796 per person living with dementia [39]. Reliance on informal care is high globally, accounting for 50% of societal costs [39]. The findings of our study have global public policy implications of affirming that the role of ACP is associated with the advancement of care outcomes and efficiency for people living with ADRD.

The findings of our study are limited by a single urban geographic area, a single year of observation, a single non-profit organization health system and a Medicare Advantage population. Further studies with NGTC partners with different characteristics may yield different results. As we reviewed administrative data templates without personal information identification, we did not review the healthcare providers’ progress notes in the EHR. Although the factors associated with access to healthcare were not included in our study, only Medicare Advantage enrollees were collected to avoid the impact of health insurance on telehealth access; Medicaid or dual Medicare- and Medicaid-eligible individuals were restricted only to telehealth service reimbursements in audio-only and asynchronous services when our analysis was conducted [40]. The hospitalization-associated cost was generated from the charge rates of the health systems that did not provide actual reimbursement rates or reflect geographic urban/rural differences. 

## 5. Conclusions

Although the overall ACP documentation rate was low, when present, ACP documentation contributed to positive impacts on saving healthcare costs and fewer hospitalizations among patients diagnosed with ADRD. Telehealth is an efficient delivery tool for engaging in discussions about advance care preferences and documenting them, especially in an area with a provider shortage. Further geriatrics workforce training is called for to enhance the competencies of primary care providers in prioritizing ACP via telehealth in the care of those living with ADRD.

## Figures and Tables

**Table 1:** Descriptive statistics of patient characteristics.

Variables		*n* = 58	
	
		*N*	(%)
Age	Mean ± standard deviation	81.3 ± 9.7 (range 61–98)	
	−79	22	37.9
	80+	36	62.1
Race			
	Non-Hispanic White	30	51.7
	Non-Hispanic Black	9	15.5
	Hispanic	8	13.8
	Non-Hispanic Asian	7	12.1
	Other or mixed races	4	6.9
Gender			
	Male	24	41.4
	Female	32	55.3
	Other	2	3.6
Charlson Comorbidity Index			
	Mean ± standard deviation	3.9 ± 2.7 (range 2–11)	

**Table 2:** Hospitalization-associated utilization between ADRD patients with and without ACP documentation.

Mean (Standard Deviation)	Without ACP Documentation (*n* = 58)	With ACP Documentation (*n* = 58)	*p*
Number of hospitalizations	1.27 (0.67)	0.74 (0.31)	<0.01
Number of 90-day re-hospitalizations	0.31 (0.18)	0.16 (0.06)	<0.01

**Table 3:** Hospitalization-associated cost comparison between ADRD patients with and without ACP documentation.

Cost Estimate (USD)	Without ACP Documentation (*n* = 58)	With ACP Documentation (*n* = 58)	*p*
Mean (Standard Deviation)	200,148 (82,061)	149,722 (80,850)	<0.01

**Table 4:** Trends in ACP billing rates of ADRD patients before and after implementation of telehealth primary care.

	Before Telehealth Primary Care (*n* = 473)	After Telehealth Primary Care (*n* = 473)	*χ* ^2^	*p*
99497	4 (0.8%)	58 (12.3%)	50.3	<0.001

## Data Availability

Not applicable, as all data are presented in the article.
